# Removal of sulfur by adding zinc during the digestion process of high-sulfur bauxite

**DOI:** 10.1038/s41598-017-17499-4

**Published:** 2017-12-07

**Authors:** Zhanwei Liu, Wenhui Ma, Hengwei Yan, Keqiang Xie, Dunyong Li, Licong Zheng, Pengfei Li

**Affiliations:** State Key Laboratory of Complex Nonferrous Metal Resources Clean Utilization/National Engineering Laboratory for Vacuum Metallurgy, Kunming University of Science and Technology, Kunming, 650093 China

## Abstract

This paper proposes a novel approach to sulfur removal by adding zinc during the digestion process. The effects of zinc dosage on the concentrations of different valence sulfur in sodium aluminate solution were investigated at length to find that high-valence sulfur (S_2_O_3_^2−^, SO_3_^2−^, SO_4_^2−^) concentration in sodium aluminate solution decreases, but the concentration of the S^2−^ in the sodium aluminate solution increases as zinc dosage increases. This suggests that zinc can react with high-valence sulfur to generate S^2−^ at digestion temperature, which is consistent with our thermodynamic calculation results. In this study, as zinc dosage increases, sulfur digestion rate decreases while sulfur content in red mud markedly increases when zinc dosage was below 4%; the digestion rates of sulfur and sulfur content in red mud remains stable when zinc dosage was above 4%; the alumina digestion rate, conversely, increased slightly throughout the experiment. This suggests that high-valence sulfur in sodium aluminate solution can be converted to S^2−^ and then enter red mud to be removed completely by adding zinc during the digestion process.

## Introduction

Extensive and rapid developments in the alumina industry have made bauxite resources increasingly scarce. China possesses over 800 million tons of diasporic high-sulfur bauxite, mainly in Henan, Guizhou, and Chongqing provinces^1^; if effective methods of removing the sulfur of this bauxite are made available, it is highly valuable.

During the Bayer process, the sulfur in high-sulfur bauxite first enters the solution in the form of S^2−^, then the S^2−^ is gradually oxidized into various forms of high-valence.

Sulfur (S_2_O_3_^2−^, SO_3_^2−^, SO_4_^2−^). The most negative effects of sulfur’s presence in the Bayer process are as follows^2,3^: 1) Na_2_S can react with Fe_2_O_3_, and Na_2_S_2_ can react with Fe(OH)_2_ to form Na_2_[FeS_2_(OH)_2_]·2H_2_O, which is much more soluble in sodium aluminate liquor; this increases the iron content in alumina products. 2) Na_2_S_2_O_3_ can react with Fe to form Na_2_S, Na_2_SO_3_, and Fe(OH)_2_, which destroy the steel oxide film and accelerate the corrosion of equipment. 3) The sulfur in the Bayer process is eventually converted to Na_2_SO_4_, which increases alkali consumption. 4) Once the content of Na_2_SO_4_ in spent liquor reaches a certain level, it can precipitate in the form of Na_2_CO_3_·2Na_2_SO_4_; the double salt can then scale the evaporator and interior digester, which impact industrial production.

To date, relatively few researchers have investigated sulfur removal in the alumina production process because high-sulfur bauxite is seldom used in alumina production outside of China, but there are many studies of sulfur removal in other fields^4–11^. Recent years have seen a few relevant studies in China. The methods of sulfur removal can be mainly divided into flotation desulfurization^12–15^, roasting desulfurization^16–18^, bioleaching desulfurization^19,20^, electrolysis desulfurization^21–24^, wet oxidation desulfurization^25–27^, desulfurization by precipitators^28–32^ (such as barium salts, zinc oxide, lime, *et al*.). These methods have their own advantages and disadvantages, they have not been widely applied in industry. Beneficiation desulfurization prevents most of the sulfur in bauxite from contaminating the alumina production process, but sulfur in the concentrate remains problematic, requiring further desulfurization throughout the process. Wet oxidation desulfurization does not introduce any new impurities into the liquor, and the necessary materials are cheap and readily available, but some amount of hydrogen is produced during wet oxidation, so there are safety risks inherent to the process, further, discharge and causticization increase the production cost. Desulfurization efficiency of desulfurization by precipitators is high, but only a form of sulfur was removed.

Large swaths of China’s high-sulfur bauxite reserves are unable to be developed or utilized due to the lack of appropriate deep desulfurization method for alumina production. This paper proposes a new method of sulfur removal in which zinc is supplied to prevent S^2−^ from oxidizing into S_2_O_3_^2−^, SO_3_^2−^, and SO_4_^2−^ in the sodium aluminate solution, which forces S^2−^ into red mud to decrease the concentration of S^2−^, S_2_O_3_^2−^, SO_3_^2−^, SO_4_^2−^, and total sulfur in the liquor. As opposed to other methods of sulfur removal, this method does not need discharge or causticization processes to remove the sulfate, and all forms of sulfur in liquor can be removed. As discussed below, removal of sulfur via the zinc addition in liquor was investigated to provide a theoretical and technical basis for the effective utilization of high-sulfur bauxite.

## Results and Discussions

### Effects of zinc dosage on the concentrations of different valence sulfur in liquor

**Figure 1:**
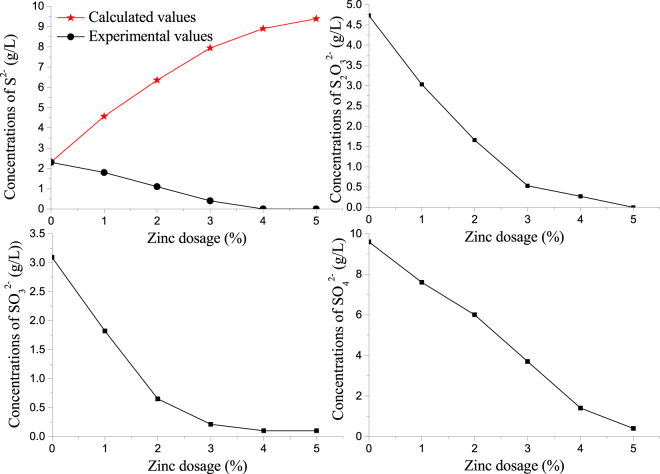
Effects of zinc dosage on the concentrations of different valence sulfur in sodium aluminate solution.

Zinc dosage was varied between 0 and 5% through a series of digestion experiments at temperature of 533 K (260 °C), digestion time of 60 min, lime dosage of 13%, digestion liquor α_k_ (molar ratio of Na_2_O_k_ to Al_2_O_3_) of 1.42. The effects of zinc dosage on the concentrations of different valence sulfur in sodium aluminate solution are shown in Fig. 1.

**Figure 2:**
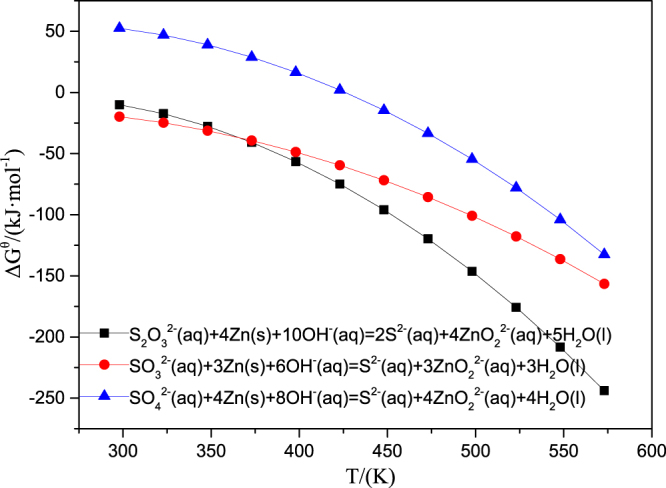
The diagram of standard Gibbs free energy with temperature of the reactions of zinc with high-valence sulfur in sodium aluminate solution.

Figure 1 shows that the concentration of the high-valence sulfur (S_2_O_3_^2−^, SO_3_^2−^, SO_4_^2−^) in the sodium aluminate solution decreased as zinc dosage increased, while the calculated values of the S^2−^ concentration in the solution increased notably with the increase of zinc dosage. This suggests that zinc can react with high-valence sulfur to generate low-valence sulfur at the digestion temperature, which is consistent with our thermodynamic calculations (as shown in Fig. 2). Calculated values of the S^2−^ concentration are attained according to reactions as follows:

1 $${{\rm{S}}}_{2}{{{\rm{O}}}_{3}}^{2-}({\rm{a}}{\rm{q}})+4{\rm{Z}}{\rm{n}}({\rm{s}})+{10{\rm{O}}{\rm{H}}}^{-}({\rm{a}}{\rm{q}})={2{\rm{S}}}^{2-}({\rm{a}}{\rm{q}})+{{4{\rm{Z}}{\rm{n}}{\rm{O}}}_{2}}^{2-}({\rm{a}}{\rm{q}})+{5{\rm{H}}}_{2}{\rm{O}}({\rm{l}})$$

2 $${{{\rm{S}}{\rm{O}}}_{3}}^{2-}({\rm{a}}{\rm{q}})+3{\rm{Z}}{\rm{n}}({\rm{s}})+{6{\rm{O}}{\rm{H}}}^{-}({\rm{a}}{\rm{q}})={{\rm{S}}}^{2-}({\rm{a}}{\rm{q}})+{{3{\rm{Z}}{\rm{n}}{\rm{O}}}_{2}}^{2-}({\rm{a}}{\rm{q}})+{3{\rm{H}}}_{2}{\rm{O}}({\rm{l}})$$

3 $${{{\rm{S}}{\rm{O}}}_{4}}^{2-}({\rm{a}}{\rm{q}})+4{\rm{Z}}{\rm{n}}({\rm{s}})+{8{\rm{O}}{\rm{H}}}^{-}({\rm{a}}{\rm{q}})={{\rm{S}}}^{2-}({\rm{a}}{\rm{q}})+{{4{\rm{Z}}{\rm{n}}{\rm{O}}}_{2}}^{2-}({\rm{a}}{\rm{q}})+{4{\rm{H}}}_{2}{\rm{O}}({\rm{l}})$$

It also can be seen from Fig. 1 that in this experiment, as zinc dosage increased, the concentration of the S^2−^ in the sodium aluminate solution decreased notably when zinc dosage was below 3%; the concentration of the S^2−^ remains stable when zinc dosage was above 3%; the experimental values of the S^2−^ concentration is lower than calculated values throughout the experiment because the S^2−^ converted in solution entered into red mud; when zinc dosage was 5%, the S^2−^ and S_2_O_3_^2−^ in liquor were removed completely, the SO_3_^2−^ and SO_4_^2−^ were removed nearly completely. So the sulfur in sodium aluminate solution can be effectively removed by adding zinc in the digestion process.

### Thermodynamic calculation

The standard Gibbs free energy of the reactions of zinc with high-valence sulfur (S_2_O_3_^2−^, SO_3_^2−^, SO_4_^2−^) was calculated using Factsage 7.0 software, the results are shown in Fig. 2.

As can be seen from Fig. 2, the Δ*G* ^θ^ of the reactions of zinc with S_2_O_3_^2−^, SO_3_^2−^ were all negative value in the temperature range of 298~573 K (25~300 °C), the Δ*G* ^θ^ of the reaction of zinc with SO_4_^2−^ was negative value when the temperature above 448 K (175 °C). This suggests that zinc can react with high-valence sulfur to generate S^2−^ at digestion temperature, because digestion temperature of high-sulfur bauxite is higher than 513 K (240 °C) in industrial production.

The more negative the Δ*G* ^θ^ value, the more favorable the reaction is, so S_2_O_3_^2−^ is easiest to be reduced by zinc, then SO_3_^2−^, SO_4_^2−^ is most difficult to be reduced at temperature of 533 K (260 °C), this explains why S_2_O_3_^2−^ in liquor were removed completely, the SO_3_^2−^ and SO_4_^2−^ were removed nearly completely when zinc dosage was 5% (as shown in Fig. 1).

### Effects of zinc dosage on digestion rates of alumina and sulphur

**Figure 3:**
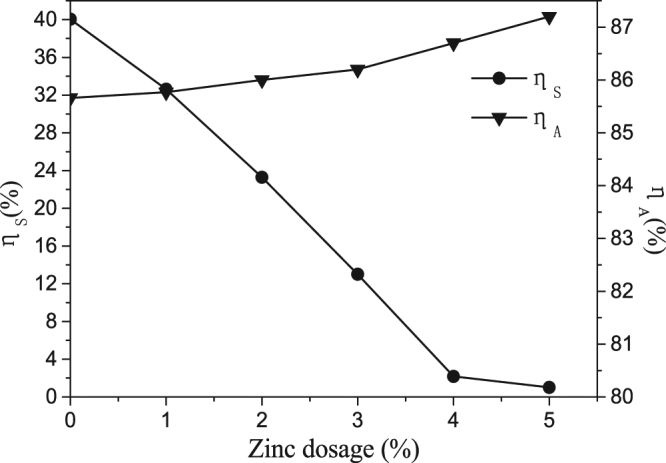
Effects of zinc dosage on digestion rates of alumina and sulfur.

Again, six different zinc dosages (between 0% and 5%) were tested under the same other conditions described above. The Effects of zinc dosage on the digestion rates of alumina and sulfur are shown in Fig. 3.

It can be seen from Fig. 3 that as zinc dosage increased, the digestion rates of sulfur decreased obviously when zinc dosage was below 4%; when zinc dosage was above 4%, the digestion rates of sulfur remains stable. While the alumina digestion rate increased slightly throughout the experiment. Zinc in sodium aluminate solution exists mainly in the form of ZnO_2_^2−^, the solubility product constant of ZnS (2 × 10^−24^) is very low, so it is difficult to decompose in sodium aluminate solution, the S^2−^ can react with Zn^2+^ easily to generate ZnS^33,34^, the more S^2−^ in liquor, the easier ZnO_2_^2−^ produce to Zn^2+^. As a reductant, the zinc reacted with high-valence sulfur to generate S^2−^, and then the S^2−^ reacted with Zn^2+^ to generate ZnS went into red mud, so the sulfur in liquor was removed. The reactions are as follows:

4 $${{{\rm{Z}}{\rm{n}}{\rm{O}}}_{2}}^{2-}+{2{\rm{H}}}_{2}{\rm{O}}={{\rm{Z}}{\rm{n}}}^{2+}+{4{\rm{O}}{\rm{H}}}^{-}$$

5 $${{\rm{Z}}{\rm{n}}}^{2+}+{{\rm{S}}}^{2-}={\rm{Z}}{\rm{n}}{\rm{S}}$$

**Figure 4:**
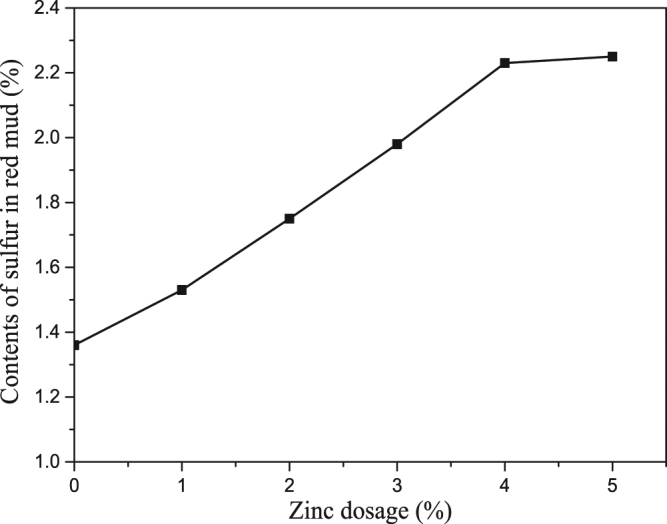
Effects of zinc dosage on content of sulfur in red mud.

The sulfur content increased in red mud as zinc dosage increased (as shown in Fig. 4), while the digestion rates of sulfur decreased. When zinc dosage was above 4%, the sulfur in liquor was nearly removed.

### Effects of zinc dosage on content of sulfur in red mud

**Figure 5:**
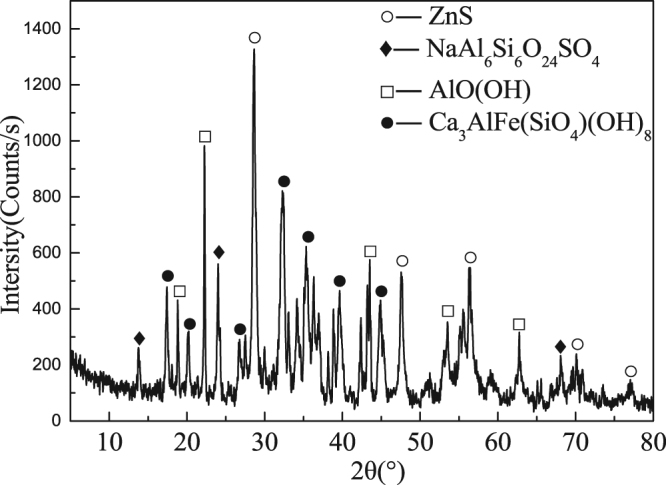
X-ray diffraction pattern of red mud.

The contents of sulfur in red mud described above are shown in Fig. 4. The X-ray diffraction pattern of red mud is shown in Fig. 5, where digestion temperature is 533 K (260 °C), digestion time is 60 min, and zinc dosage is 5%.

It can be seen from Fig. 4 that when zinc dosage was below 4%, the sulfur content increased substantially in the red mud as zinc dosage increased; the sulfur content remains stable when zinc dosage was above 4%.

The results shown in Figs 3 and 4 altogether indicate that sulfur in sodium aluminate solution can be effectively removed by adding zinc in the digestion process.

We also find ZnS in red mud, as shown in Fig. 5.

### Effects of zinc dosage on the concentrations of iron in sodium aluminate solution

**Figure 6:**
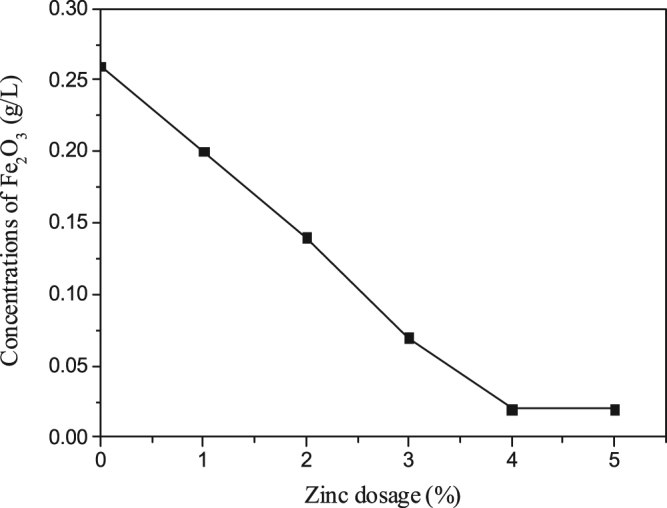
Effects of zinc on the concentrations of iron in liquor.

Again, six different zinc dosages (between 0% and 5%) were tested under the same other conditions described above. The Effects of zinc dosage on the concentrations of iron in sodium aluminate solution are shown in Fig. 6.

It can be seen from Fig. 6 that when zinc dosage was below 4%, the concentration of Fe_2_O_3_ decreased substantially as zinc dosage increased; the concentration of Fe_2_O_3_ remains stable when zinc dosage was above 4%. So iron in sodium aluminate solution was also removed, when sulfur in liquor was removed by adding zinc during the digestion process.

### Effects of zinc dosage on absorbance of liquor

**Figure 7:**
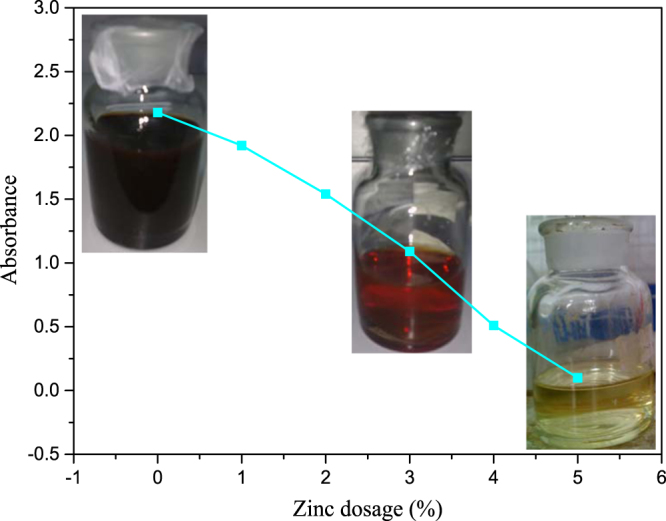
Absorbance of liquor as a function of zinc dosage.

Again, six different zinc dosages (between 0% and 5%) were tested under the same other conditions described above. The effects of zinc dosage on absorbance of liquor are shown in Fig. 7. Sulfur removal rate is measured by observing the absorbance change of liquor, represented by color change. Absorbance was measured at 578 nm in a 4 cm cell in this study.

It can be seen from Fig. 7 that the absorbance of liquor decreased as zinc dosage increased. This suggests that the color of sodium aluminate liquor fades notably.

It can be seen visually from Fig. 7 that the colours of digestion liquors change from opaque to transparency with the increase of zinc dosage, the colours of digestion liquor is the same as that of normal digestion liquor in the Bayer process when zinc dosage is 5%, this means that the addition of zinc in liquor can make the colour of sodium aluminate solution fade noticeably.

The higher the sulfur concentration in sodium aluminate solution, the deeper the colour of solution^35^. So, it can be seen from Fig. 7 that the sulfur in sodium aluminate solution can be effectively removed by adding zinc in the digestion process.

### Mechanism of sulfur removal

**Figure 8:**
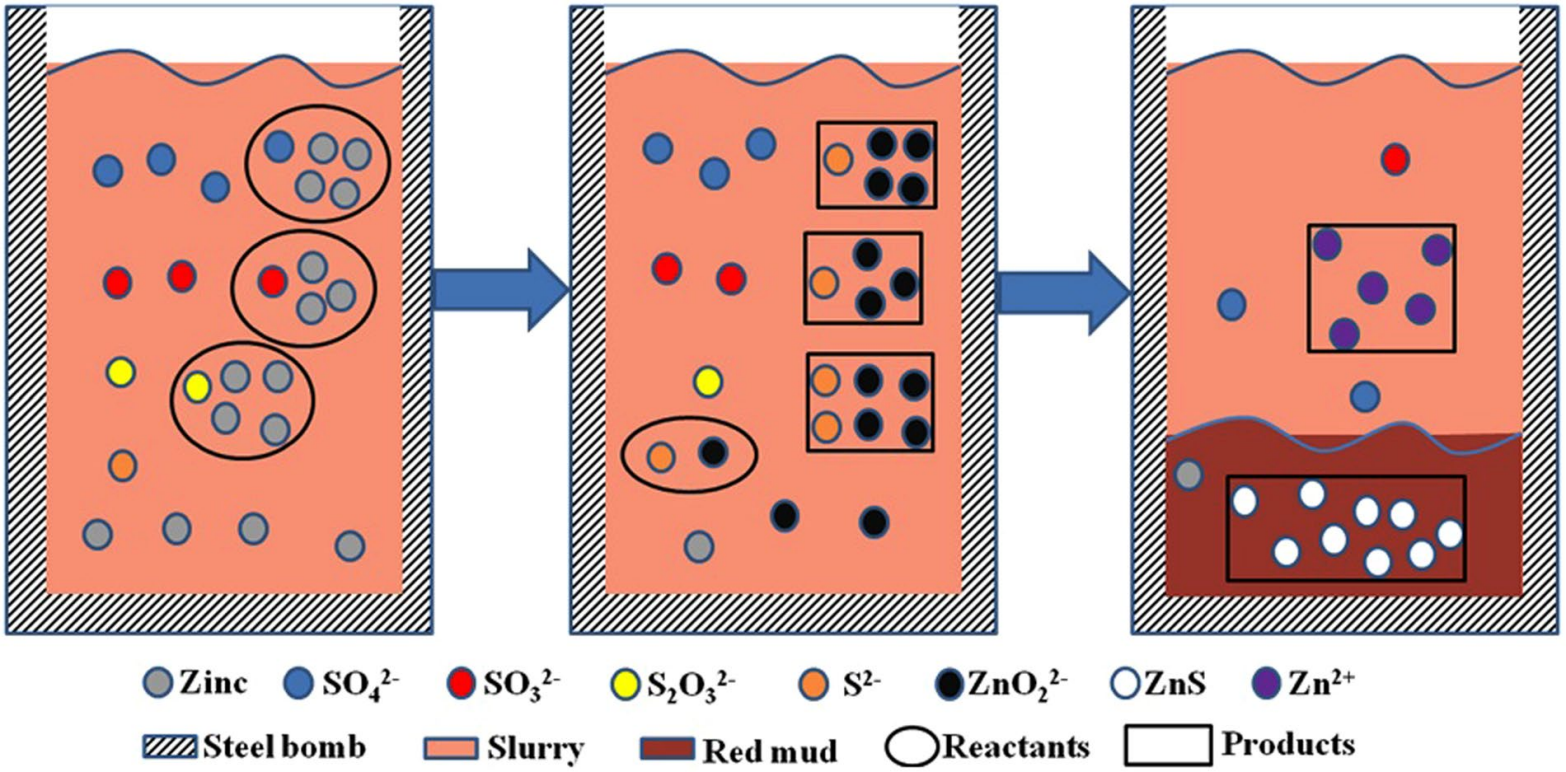
Schematic illustration of sulfur removal mechanism by adding zinc in the sodium aluminate solution.

Based the above result and discussion, we propose the following mechanism of sulfur removal (shown in Fig. 8). As a reductant, the zinc reacted with high-valence sulfur (S_2_O_3_^2−^, SO_3_^2−^, SO_4_^2−^) to generate low-valence sulfur (S^2−^), zinc in sodium aluminate solution mostly in the form of ZnO_2_^2−^, the S^2−^ promotes ZnO_2_^2−^ to produce Zn^2+^, and then the S^2−^ reacted with Zn^2+^ to generate ZnS went into red mud. As a reductant and precipitator, the zinc can remove sulfur completely in sodium aluminate solution during the digestion process.

## Conclusions

The high-valence sulfur in sodium aluminate solution can be converted to S^2−^ and entered into red mud in the form of ZnS by adding zinc during the digestion process. In this study, as zinc dosage increases, sulfur digestion rate decreases while sulfur content in red mud markedly increases when zinc dosage was below 4%; the digestion rates of sulfur and sulfur content in red mud remains stable when zinc dosage was above 4%; the alumina digestion rate, conversely, increased slightly throughout the experiment. Considering both production cost and desulfurization efficiency, the optimum zinc dosage was determined to be 4%. So the sulfur in sodium aluminate solution can be effectively removed by adding zinc in the digestion process, which provide a theoretical basis for the effective removal of sulfur in alumina production process.

## Materials and Method

### Materials

**Figure 9:**
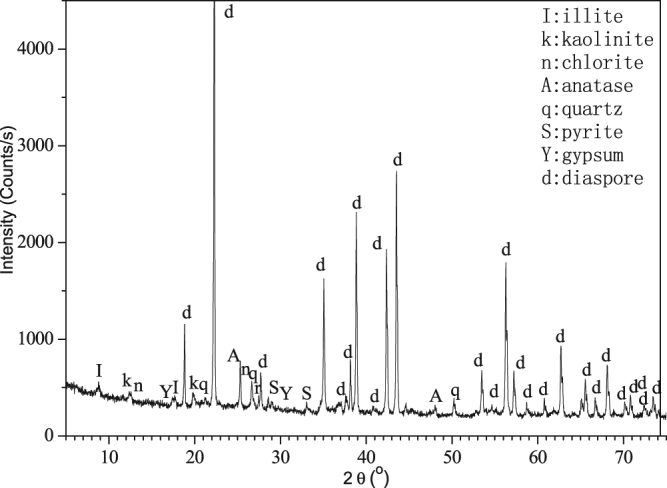
X-ray diffraction pattern of high-sulfur bauxite.

**Figure 10:**
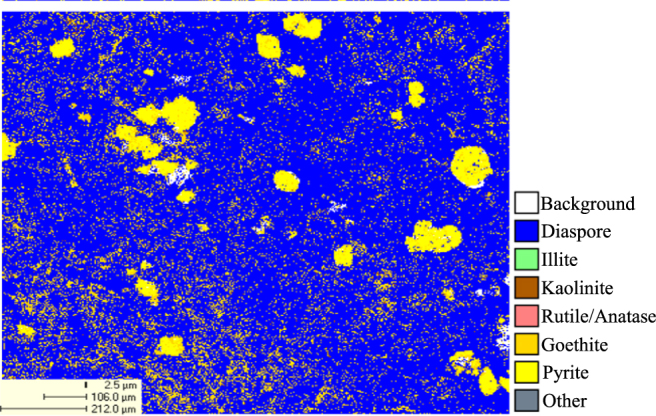
QEMSCAN image of the high sulfur bauxite.

The high-sulfur bauxite used in this experiment was obtained from Zunyi mining area in China. The chemical components of mineral samples are shown in Table 1. The X-ray diffraction pattern of the mineral is shown in Fig. 9. The QEMSCAN image of the mineral is shown in Fig. 10.

**Table 1:** Chemical components of high-sulfur bauxite.

Chemical components	Amount (Wt%)
Al_2_O_3_	63.99
SiO_2_	8.12
Fe_2_O_3_	6.66
TiO_2_	2.86
K_2_O	1.23
Na_2_O	0.006
CaO	0.22
MgO	2.95
S_Total_	2.05
C_Total_	0.42
C_Organic_	0.31

As shown in Figs 9 and 10, the primary sulfur-bearing mineral is pyrite. Figure 10 also demonstrates that pyrite was a granular aggregate distribution, particle size is 10~100 μm, and goethite was infection distribution in pyrite around. During the Bayer process of alumina production, the sulfur in high-sulfur bauxite first enters the solution in the form of S^2−^, then the S^2−^ is gradually oxidized into various forms of S_2_O_3_^2−^, SO_3_^2−^, and SO_4_^2−^.

The chemical components of the alkali solution used in the experiment are listed in Table 2.

**Table 2:** Chemical components of evaporation spent liquor of Zunyi alumina refinery.

Chemical components	Concentration (g/L)
Na_2_O_T_	248.11
Al_2_O_3_	120.2
Na_2_O_k_	216
Na_2_S	0.24
Na_2_S_2_O_3_	4.67
Na_2_SO_3_	2.84
Na_2_SO_4_	6.58

Note: Na_2_O_T_—total soda (as Na_2_O), Na_2_O_k_—caustic soda (as Na_2_O).

### Method

**Figure 11:**
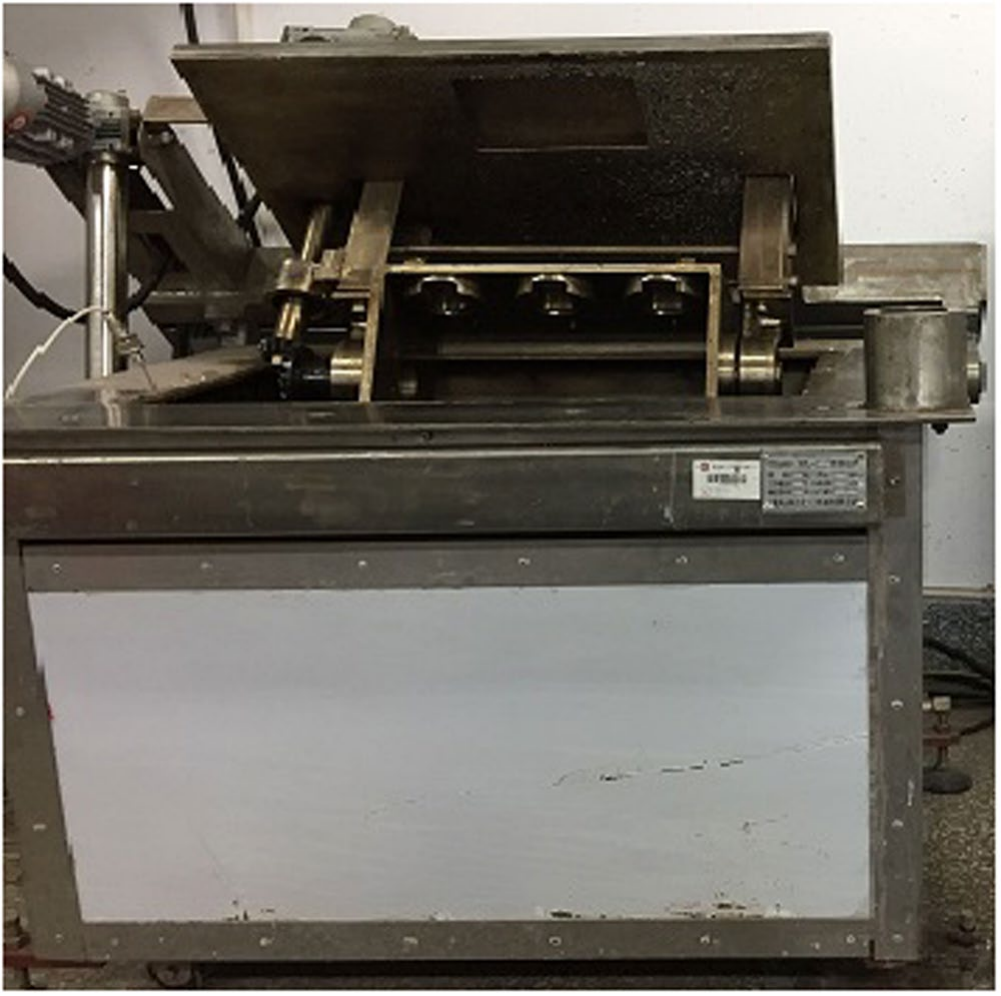
Digestion experiment equipment.

Digestion experiments on high-sulfur bauxite were conducted in a XYF-6 digester, as shown in Fig. 11, which heated by molten salts.

The mineral sample and evaporation spent liquor according to a certain proportion were placed in a 100 ml stee1 bomb which was sealed in the experiment. The digester was heated to 533 K (260 °C) and held for 5 min, then the steel bomb was placed inside and the digestion process was run 60 min. The sodium aluminate solution was then filtered and the Al_2_O_3_, Na_2_O_k_, Na_2_O_T_, and different valence sulfur (S^2−^, S_2_O_3_^2−^, SO_3_^2−^, and SO_4_^2−^) in the filtrate were chemically analyzed^36^. The dried red mud was sampled and observed with an X-ray fluorescence analyzer and carbon-sulfur analyzer.

The digestion rate of alumina (η_A_) was calculated as follows:

6 $${\eta }_{A}=\frac{{(A/S)}_{ore}-{(A/S)}_{mud}}{{(A/S)}_{ore}}\times 100{\rm{ \% }}$$

(A/S)_ore_ Mass ratio of alumina to silica in raw ore

(A/S)_mud_ Mass ratio of alumina to silica in mud

The digestion rate of sulfur (η_S_) was calculated as follows:

7 $${\eta }_{S}=\frac{{S}_{ore}-\frac{{F}_{ore}}{{F}_{mud}}{S}_{mud}}{{S}_{ore}}\times 100{\rm{ \% }}$$

S_ore_ Mass percentage of sulfur in raw ore (%)

S_mud_ Mass percentage of sulfur in mud (%)

F_ore_ Mass percentage of iron in raw ore (%)

F_mud_ Mass percentage of iron in mud (%)
